# Proximate causes of the red face of the bald uakari monkey (*Cacajao calvus*)

**DOI:** 10.1098/rsos.150145

**Published:** 2015-07-29

**Authors:** P. Mayor, J. Mamani, D. Montes, C. González-Crespo, M. A. Sebastián, M. Bowler

**Affiliations:** 1 Department de Sanitat i Anatomia Animals, Faculty of Veterinary, Universitat Autònoma de Barcelona, Bellaterra, Barcelona, Spain; 2 YAVACUS, Yavarí Conservación y Uso Sostenible, Iquitos, Perú; 3 Laboratory of Pathology, Faculty of Veterinary, Universidad Peruana Cayetano Heredia, Lima, Perú; 4 Departament de Medicina i Cirurgia Animals, Servei d' Ecopatologia de Fauna Salvatge (SEFaS), Faculty of Veterinary, Universitat Autònoma de Barcelona, Bellaterra, Barcelona, Spain; 5 SAMAI, Iquitos, Perú; 6 San Diego Zoo Global, Institute for Conservation Research, Escondido, CA, USA; 7 School of Psychology and Neuroscience, University of St Andrews, St Andrews, UK

**Keywords:** uakari, *Cacajao calvus*, face, coloration, health indicator

## Abstract

In social species, such as primates, facial appearances transmit a variety of social signals. Although it is suggested that the intense red colour of the face of the bald uakari monkey might be an indicator of health, this hypothesis still has not been verified. This study describes the histological structure of the skin of the face in the bald uakari, compared with other non-red neotropical primates, to better understand the maintenance of its colour. The facial skin of the bald uakari monkey is characterized by a thinner epidermis, absence of melanin pigments and a high density of vascular capillaries that spread below the epidermis. These vascular capillaries are larger and more tortuous than in other neotropical primates. The skin of the face of the bald uakari monkey allows a direct external assessment of haematological status, suggesting that the colour of the face would be an honest indicator of health, but could also signal sexual or behavioural states.

## 1. Introduction

Primates process information signalled by faces more rapidly than other stimuli [1], and within the order, a wide range of phenotypes for function, location, colour and shape create a high diversity of facial colour patterns [2]. While the facial colour pattern is used primarily for species recognition, secondary colour variations serve to assess individual identity [2,3], providing rich sources of information about behaviour and condition [4] that are essential for social interactions [5]. Thus, social functions, such as female mate preference, may be the primary designers of the evolution of primate facial colour patterns across species [6].

Primates exhibit a vivid and colourful array of visual signals [6,7]. Sometimes, as in baboons [8] and chimpanzees [9], these are brightly coloured external genital organs, and in other species, such as Japanese macaques [10], mandrills [4] and rhesus macaques [11], facial coloration acts as a visual signal. In these species, facial redness varies throughout the ovarian cycle and contains information about the timing of the fertile phase [10–12]. Additionally, rhesus macaque females are more attracted by male reddish faces [3,13].

The facial redness is influenced by the degree of epidermal blood flow through action on oestrogen-dependent receptors in the hairless face [14]. These oestrogen receptors are developed after puberty [15] and are only present in sexual skin [16]. Whereas in females, facial redness is directly linked to oestrogen [16]; in males, facial coloration is indirectly linked to testosterone, which is aromatized by aromatase to oestrogen [17]. Oestrogen regulates variation in blood flow reducing systemic vascular resistance and increasing the cardiac output [18], resulting in changes of the red colour of the skin [17]. Although changes in skin redness are apparently linked to variation in blood flow and oxygenation [19], the mechanism for this is unknown.

The reasons underlying female preference for ornamented males also remain a matter of debate [8,20,21]. Handicap theories of mate choice propose that only individuals of ‘superior quality’ will be able to express exaggerated secondary sexual ornaments, such as bright or intense coloration [22]. In that sense, the Hamilton–Zuk hypothesis suggests that secondary sexual ornaments may reliably reflect ability to resist parasites by revealing current health status [23].

The bald uakari monkey (*Cacajao calvus*), is a vulnerable [24] ‘red-faced’ monkey emblematic of Amazonian flooded forests. Ayres [25] suggested that the red face is a signal by which individuals can assess the health status of potential mates, enabling them to select mates with low parasite burdens and, therefore, good resistance to parasites. Although several authors have observed that the facial colour in dead and sick uakaris turns pale [26–28], the hypothesis still has not been verified and little is known about the developmental and mechanistic processes that result in the red colour of the uakari face. This study aims to describe the histology of the facial skin in the bald uakari monkey to better understand the proximate causes for changes in intensity (luminance or redness) in the red face of this species. We hypothesized that the expression of the red skin colour in the bald uakari is owing to the histological structure of the facial skin that signals the flow of blood.

## 2. Methods

We collected skin samples from two deceased Peruvian red uakari monkeys (*Cacajao calvus ucayalii*), a red-furred subspecies of the bald uakari, from the Pilpintuwasi Amazon Animal Orphanage Centre, during their autopsies. Additionally, between 2012 and 2014, we collected the skin samples from different primates that were hunted as part of the normal subsistence hunting of indigenous people from the community of Nueva Esperanza in the Yavarí-Mirín River. Samples were collected from two Peruvian red uakari monkeys (*Cacajao calvus ucayalii*), two Poeppig's woolly monkeys (*Lagothrix poepigii*), two monk sakis (*Pithecia monachus*), two brown capuchin monkeys (*Sapajus macrocephalus*) and one howler monkey (*Alouatta seniculus*). No animals were killed specifically for the research, and hunters were never paid to collect samples.

**Figure 1.**
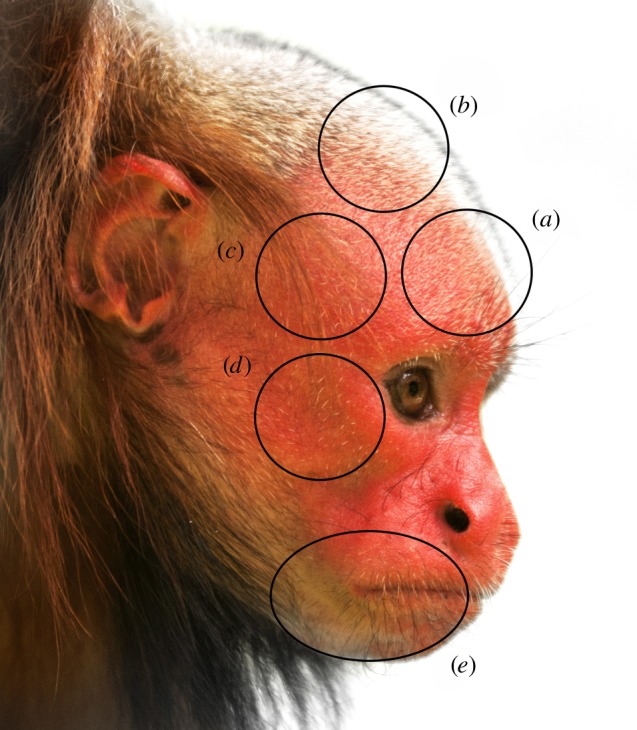
Facial regions studied in the bald uakari monkey and other neotropical primates: (*a*) frontal region, (*b*) parietal region, (*c*) temporal region, (*d*) zygomatic region, and (*e*) mandible region.

We collected the skin samples from five anatomical regions (parietal, temporal, frontal, mandible and zygomatic facial regions; figure 1) from all sampled primates. Additionally, from red uakari specimens, we collected a skin sample of the thoracic and lumbar region in order to characterize the differences between different body regions.

We dehydrated skin samples, embedded them in paraffin wax, sectioned at 3 μm sections and stained by haematoxylin and eosine (H&E). We measured epidermal thickness, and density and size of vascular capillaries using a light microscope. Density of vascular capillaries was calculated by counting the number of capillaries at the superficial interface between the dermis and epidermis. The size of vascular capillaries was measured in transverse sections by placing callipers on the external surface. All variables were determined in five randomly selected areas in each skin region.

First, we calculated the average measurements of epidermis thickness and vascular density in the studied facial areas of each individual, and we compared those values using a generalized linear model. Second, we compared epidermis thickness and vascular density in each facial region between and within bald uakaris and other primate species, using linear-mixed models and Tukey tests. Within the bald uakaris, we also tested differences between vascular width, length and area. The five pseudo-replicate epidermal measurements in each facial region were nested to correspondent individual between bald uakaris and other primate species. Epidermal measurements were dependent variables, the anatomical region and group were fixed effects, and individual was random effect. Statistical analyses were performed using R-Studio version 0.98.1062 2009–2013 (RStudio, Inc. with lme4 Package and Deducer JRG version 1,7–9, 2003–2011 RoSuDa, Univ. Augsburg).

## 3. Results

**Figure 2.**
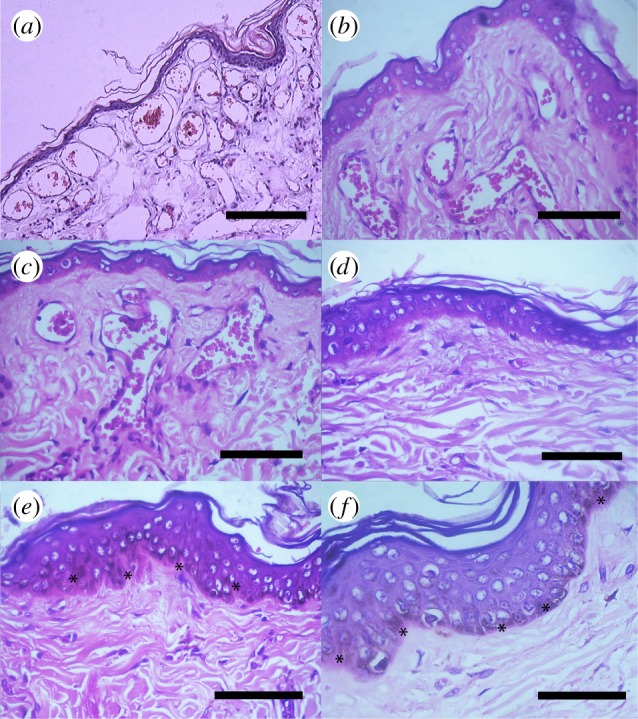
Sections of the skin from different anatomical regions in the bald uakari and other neotropical primates: (*a*) skin of the frontal region in the uakari monkey, H&E (sale bar, 250 μm), (*b*) skin of the temporal region in the uakari monkey, H&E (scale bar, 50 μm), (*c*) skin of the zygomatic region in the uakari monkey, H&E (scale bar, 50 μm), (*d*) skin of the lumbar region in the uakari monkey, H&E (scale bar, 50 μm), (*e*) skin of the zygomatic region in the brown capuchin monkey, H&E (scale bar, 50 μm), and (*f*) skin of the mandible region in the howler monkey, H&E (scale bar, 40 μm). The facial skin in the bald uakari is characterized by a thinner epidermis, absence of melanin granules and a higher density and larger vascular capillaries compared with other anatomical regions in the uakari and compared with facial regions in other neotropical primates.

The statistical model classified all studied specimens in two groups: the four Peruvian bald uakari monkeys versus the other species (epidermis thickness *F*_1,9*t*_:1396.3, *t*-value=−37.37, *p*<0.00001; vascular density *F*_1,9_:847.77, *t*-value=29.12, *p*<0.00001). The facial skin of the bald uakari was characterized by a thinner epidermis, a higher density of vascular capillaries in the horizontal upper plexus and a larger luminal surface of vascular capillaries compared with those in non-red-faced monkeys (table 1 and figure 2). Additionally, whereas granules of pigment were not observed in the epidermis in the facial skin of the bald uakari, the facial skin of non-red coloured monkeys showed numerous melanin granules.

**Table 1.** Epidermal features in different body regions in four bald uakaris (*Cacajao calvus*) and seven non-red neotropical monkeys. (n.a., non analysed; collapsed, vascular capillaries occluded. The lumen of collapsed vascular capillaries was not collected.)

		bald uakari	non-red species	
epidermal features		(*n*=4)	(*n*=7)	
epidermal thickness (μm)	temporal region	12.91±0.33^ab1^	28.70±1.64^a2^	*p*<0.00001, t_1,36_=−20.05
	parietal region	13.31±0.43^a1^	30.98±3.48^a2^	*p*<0.00001, *t*_1,36_=−10.88
	frontal region	8.78±0.28^c1^	30.04±0.82^a2^	*p*<0.00001, t_1,36_=−49.39
	mandible region	14.64±0.35^d1^	40.23±3.48^b2^	*p*<0.00001, *t*_1,36_=8.79
	zygomatic region	11.88±1.22^b1^	40.22±2.68^b2^	*p*<0.00001, *t*_1,36_=−19.68
	thoracic region	36.10±0.58^e1^	n.a.	
	lumbar region	13.08±0.75^a1^	n.a.	
		*F*_5,18_=808.4;	*F*_5,23_=30.2;	
		*p*<0.0001	*p*<0.0001	
density of vascular capillaries (n°/mm^2^)	temporal region	34.64±1.14^a1^	14.07±2.55^2^	*p*<0.00001, *t*_1,9_=15.20
	parietal region	37.10±0.66^a1^	13.32±1.89^2^	*p*<0.00001, *t*_1,9_=25.57
	frontal region	53.93±4.18^b1^	13.54±1.71^2^	*p*<0.00001, *t*_1,9_=23.10
	mandible region	37.27±1.45^a1^	13.14±1.27^2^	*p*<0.00001, *t*_1,9_=29.01
	zygomatic region	60.48±0.57^b1^	14.29±2.73^2^	*p*<0.00001, *t*_1,9_=−19.68
	thoracic region	17.23±1.10^c^	n.a.	
	lumbar region	17.01±1.01^c^	n.a.	
		*F*_5,18_=352.4;	*F*_5,23_=3.5;	
		*p*<0.0001	*p*<0.061	
lumen of vascular capillaries (μm^2^)	temporal region	4118.80±200.46^a^	collapsed	
	parietal region	4196.38±296.92^a^	collapsed	
	frontal region	3182.63±92.77^b^	collapsed	
	mandible region	2371.17±27.95^c^	collapsed	
	zygomatic region	2571.45±64.32^c^	collapsed	
	thoracic region	373.26±11.30^d^	n.a.	
	lumbar region	234.31±6.55^d^	n.a.	
		*F*_5,18_=699.1;		
		*p*<0.0001		

^a,b,c,d,e^Values appearing in rows with different superscripts are different (*p*<0.05).

^1,2^Numbers appearing in columns with different superscript are different (*p*<0.05).

In the bald uakari, epidermal differences were also observed in different anatomical regions. The skin in facial regions had a thinner epidermis, a remarkably higher density of vascular capillaries, and more extensive and ingurgitate capillaries in the vascular plexuses compared with the skin in thoracic and lumbar body regions (tables 1 and 2). The thinnest epidermis was observed in the frontal region, the highest vascular density was observed in zygomatic and frontal regions, and the largest vascular capillaries were observed in the parietal and temporal regions (tables 1 and 2).

**Table 2.** Length and width of capillar vessels (in μm) and statistical differences of vascular capillaries in different regions in four bald uakaris (*Cacajao calvus*).

capillar vessels	length	width
temporal region	68.99±1.03^a^	40.74±1.05^a^
parietal region	106.28±3.89^b^	50.49±1.54^b^
frontal region	69.82±2.61^a^	39.24±5.31^a^
mandible region	66.31±1.85^c^	41.91±2.01^a^
zygomatic region	63.45±1.26^c^	39.70±2.51^a^
thoracic region	24.41±1.16^d^	16.43±0.29^c^
lumbar region	24.67±0.14^d^	14.03±0.37^c^
	*F*_5,18_=750,5; *p*<0.0001	*F*_5,18_=141,5; *p*<0.0001

^a,b,c,d,e^Values appearing in rows with different superscripts are different (*p*<0.05).

## 4. Discussion

The coloration of primate faces is understood to be an important aspect of intraspecific communication, providing information about the identity, behaviour and sexual and health condition of the individuals [2,4]. In concord with Hamilton & Zuk [23], the red face might be a signal by which individuals can assess the health status of potential mates, enabling them to select mates with low parasite burdens and/or good resistance to parasites [24]. However, Ayres' [24] suggestion that the intense red colour of the face in the bald uakari could be an indicator of health has not, to our knowledge been tested.

We confirmed that the skin of the face in the bald uakari is coloured, not by melanin pigments, but owing to a vascular specialization of the skin that allows the abundant and superficial blood flow. These features combine to create a prominent and highly visible surface that directly demonstrates the volume of blood in these blood vessels at any point in time. These findings are consistent with the hypothesis that the facial skin of the bald uakari acts as an external modified mucous membrane and an indicator of its haematologic status. Because most important causes for pale mucosa are bleeding, destruction or decreased production of red blood cells, haemoglobinopathies and anaemia [25]; haematologic pathologies will influence the intensity (luminance) of the face of the bald uakari. Because the mechanism used to create the red colour means that it is an honest signal that can only be given out when blood levels and blood pressure are sufficiently high, the skin is particularly suitable as an honest indicator of health [23].

Based on studies of Almeida & Deane [29] and Davies *et al.*[30], Ayres [24] hypothesized the evolutionary relationship between *Plasmodium* spp. and the red face of the uakari. However, there are other haemoparasites endemic in Amazon region, such as trypanosomes, that may also have a tenable evolutionary link with the red face. Lasry & Sheridan [27] reported paling of the face in a captive uakari with Chagas' myocarditis (*Trypanosoma cruzi*), and on the Yavari River, a remote area considered the stronghold for the Peruvian red uakari monkey [28], a high 64% (80/125) prevalence of trypanosomatids was found in primate populations, including a 100% (10 out of 10) prevalence in the uakari [31], suggesting a close relationship between the bald uakari and trypanosomatids. An early microcytic–hypochromic anaemia and severe progressive thrombocytopaenia are the most important haematological changes associated with a chronic infection of *Trypanosoma* [32] and most other haemoparasitic infections [33].

Skin colour is directly influenced by blood flow and oxygenation [6,13,19], and linked to the action on the oestrogen receptors both in males and females [17]. Variation in redness reflects variation in levels of blood oxygenation, whereas variation in luminance reflects variation in blood flow [13,34]. Blood flow and oxygenation are in turn associated with an individual's health status [35], providing a mechanism for producing the red colour that allows for rapid changes, controlled by blood changes in the monkey. Such responsive control of facial colour allows speculation that the head of the red uakari could have some significance in social communication [36], and the pale eyelids used in communication [37] support this hypothesis.

All red-faced primates live in large multimale–multifemale social groups, some of which have fission–fusion grouping patterns. The red facial skin of all primates probably evolved under multimale breeding conditions, with large group sizes, and in many cases, high fission–fusion grouping dynamics [17]. Dubuc *et al.* [13] observed that female rhesus macaques gaze longer at red male faces than pale images of the same males, suggesting that females prefer redder males. Sexual selection pressures are stronger on males because their reproductive rate is less limited by gamete production and parental investment than in females [38]. In the case of the uakari, however, the red face is present in both males and females, so if sexual selection is implicated then it acts on both sexes.

The proximate mechanism of the red face in the uakari are reduced pigmentation, and specialized epidermis and related blood vessels that mean that the facial colour is a good indicator of health. The sexual state of the monkey concomitantly also affects redness through changes in blood pressure, so social signalling hypotheses may explain the ultimate reasons behind the signal. Distinguishing between these ultimate hypotheses will require observations on the heath, sexual state and redness of bald uakaris.

## Data accessibility

All biological samples and histological preparations are deposited in the Institución Veterinario de Trópico y de Altura (IVITA) from the Universidad Nacional Mayor de San Marcos, recognized by the Servicio Forestal of the Ministry of Agriculture, Perú. Epidermal measurements are deposited in Pangaea (http://doi.pangaea.de/10.1594/PANGAEA.847702).

## References

[1] Tsao DY, Livingstone MS 2008 Mechanisms of face perception. Annu. Rev. Neurosci. 31, 411–437. (doi:10.1146/annurev.neuro.30.051606.094238) 18558862 10.1146/annurev.neuro.30.051606.094238 PMC2629401

[2] Santana SE, Alfaro JL, Alfaro ME 2012 Adaptive evolution of facial colour patterns in Neotropical primates. Proc. R. Soc. B 279, 2204–2211. (doi:10.1098/rspb.2011.2326) 10.1098/rspb.2011.2326 PMC3321701 22237906

[3] Waitt C, Little AC, Wolfensohn S, Honess P, Brown AP, Buchanan-Smith HM, Perret DI 2003 Evidence from rhesus macaques suggests that male coloration plays a role in female primate mate choice. Proc. R. Soc. Lond. B 270, S144–S146. (doi:10.1098/rsbl.2003.0065) 10.1098/rsbl.2003.0065 PMC1809959 14667364

[4] Setchell JM, Jean Wickings E, Knapp LA 2006 Signal content of red facial coloration in female mandrills (*Mandrillus sphinx*). Proc. R. Soc. B 273, 2395–2400. (doi:10.1098/rspb.2006.3573) 10.1098/rspb.2006.3573 PMC1636084 16928644

[5] Tibbetts EA, Dale J 2007 Individual recognition: it is good to be different. Trends Ecol. Evol. 22, 529–537. (doi:10.1016/j.tree.2007.09.001) 17904686 10.1016/j.tree.2007.09.001

[6] Bradley B, Mundy N 2008 The primate palette: the evolution of primate coloration. Evol. Anthropol. 17, 97–111. (doi:10.1002/evan.20164)

[7] Higham JP 2009 Primate coloration: an introduction to the special issue. Int. J. Primatol. 30, 749–751. (doi:10.1007/s10764-009-9381-y)

[8] Higham JP, MacLarnon AM, Ross C, Heistermann M, Semple S 2008 Baboon sexual swellings: information content of size and color. Horm. Behav. 53, 452–462. (doi:10.1016/j.yhbeh.2007.11.019) 18206889 10.1016/j.yhbeh.2007.11.019

[9] Deschner T, Heistermann M, Hodges K, Boesch C 2004 Female sexual swelling size, timing of ovulation and male behavior in wild West African chimpanzees. Horm. Behav. 46, 204–215. (doi:10.1016/j.yhbeh.2004.03.013) 15256310 10.1016/j.yhbeh.2004.03.013

[10] Fujita S, Sugiura H, Mitsunaga F, Shimizu K 2004 Hormone profiles and reproductive characteristics in wild female Japanese macaques (*Macaca fuscata*). Am. J. Primatol. 64, 367–375. (doi:10.1002/ajp.20086) 15580584 10.1002/ajp.20086

[11] Dubuc D, Brent LJN, Accamando AK, Gerald MS, MacLarnon A, Semple S, Heistermann M, Engelhardt A 2009 Sexual skin color contains information about the timing of the fertile phase in free-ranging *Macaca mulatta*. Int. J. Primatol. 30, 777–789. (doi:10.1007/s10764-009-9369-7)

[12] Higham JP, Brent LJN, Dubuc C, Accamando AK, Engelhardt A, Gerald MS, Heistermann M, Stevens M 2010 Color signal information content and the eye of the beholder: a case study in the rhesus macaque. Behav. Ecol. 21, 739–746. (doi:10.1093/beheco/arq047) 22475874 10.1093/beheco/arq047 PMC2892627

[13] Dubuc C, Winters S, Allen WL, Brent LJ, Cascio J, Maestripieri D, Ruiz-Lambides AV, Widdig A, Higham JP 2014 Sexually sellected skin colour is heritable and related to fecundity in a non-human primate. Proc. R. Soc. B 281, 20141602 (doi:10.1098/rspb.2014.1602) 10.1098/rspb.2014.1602 PMC4211451 25253459

[14] Baulu J 1976 Seasonal sex skin colouration and hormonal fluctuations in free-ranging and captive monkeys. Horm Behav. 7, 481–494. (doi:10.1016/0018-506X(76)90019-2) 828138 10.1016/0018-506x(76)90019-2

[15] Carlisle KS, Brenner RM, Montagna W 1981 Hormonal regulation of the sex skin in *Macaca nemestrina*. Biol. Reprod. 25, 1053–1063. (doi:10.1095/biolreprod25.5.1053) 7326298 10.1095/biolreprod25.5.1053

[16] Ozasa H, Gould KG 1982 Demonstration and characterization of the estrogen receptor in chimpanzee sex skin: correlation between nuclear receptor levels and degree of swelling. Endocrinology 111, 125–131. (doi:10.1210/endo-111-1-125) 7084107 10.1210/endo-111-1-125

[17] Rhodes L, Argersinger ME, Gantert LT, Friscino BH, Hom G, Pikounis B, Hess DL, Rhodes WL 1997 Effects of administration of testosterone, dihydrotestosterone, oestrogen and fadrozole, and aromatase inhibitor, on blood flow and sex skin colour in male rhesus macaques. J. Reprod. Fertil. 111, 51–57. 9370967 10.1530/jrf.0.1110051

[18] Williams JK, Kim YD, Adams MR, Chen M, Myers AD, Ramwell PW 1994 Effects of estrogen on cardiovascular responses of premenopausal monkeys. J. Pharmacol. Exp. Ther. 271, 671–676. (doi:0022-3565/9412712-0671$03.OO/O) 7965781

[19] Dixson AF 2012 Primate sexuality: comparative studies of prosimians, monkeys, apes, and human beings. Oxford, UK: Oxford University Press.

[20] Andersson M 1994 Sexual selection. Princeton, NJ: Princeton University Press.

[21] Kokko H, Brooks R, Jennions MD, Morley J 2003 The evolution of mate choice and mating biases. Proc. R. Soc. Lond. B 270, 653–664. (doi:10.1098/rspb.2002.2235) 10.1098/rspb.2002.2235 PMC1691281 12769467

[22] Zahavi A 1975 Mate selection: a selection for handicap. J. Theor. Biol. 53, 205–214. (doi:10.1016/0022-5193(75)90111-3) 1195756 10.1016/0022-5193(75)90111-3

[23] Hamilton WD, Zuk M 1982 Heritable true fitness and bright birds: a role for parasites? Science 218, 384–387. (doi:10.1126/science.7123238) 7123238 10.1126/science.7123238

[24] Veiga LM, Bowler M 2013 *Cacajao calvus* ssp. *ucayalii*. In IUCN 2013. IUCN Red List of Threatened Species 2008. Version 2013 www.iucnredlist.org. See http://www.iucnredlist.org. Accessed April 12, 2013.

[25] Ayres JM 1986 The white uakaris and the Amazonian flooded forests. PhD thesis Cambridge University, Cambridge, UK.

[26] Hill CA 1965 Maintenance of facial coloration in the red uakari *Cacajao rubicundus*. Int. Zoo Year B. 5, 140–141. (doi:10.1111/j.1748-1090.1965.tb01611.x)

[27] Lasry JE, Sheridan BW 1965 Chagas' myocarditis and heart failure in the red uakari *Cacajao rubicundus*. Int. Zoo Year B. 5, 182–184. (doi:10.1111/j.1748-1090.1965.tb01633.x)

[28] Bowler M 2007 The ecology and conservation of the red uakari monkey on the Yavari River, Peru. PhD thesis. University of Kent, Canterbury, UK.

[29] Almeida FB, Deane LM 1970 *Plasmodium brasilianum* encontrado em seu hospedeiro original, o macaco uacari branco, *Cacajao calvus*. Bol. Inst. Nac. Pesq. Amazonia 4, 1–9.

[30] Davies CR, Ayres JM, Dye C, Deane LM 1991 Malaria infection rate of Amazonian primates increases with body weight and group size. Funct. Ecol. 5, 655–662. (doi:10.2307/2389485)

[31] Aysanoa E 2014 Prevalence of trypanosomatids and trypanosoma cruzi in wild and captive non-human primates from Peru. In *63rd Annual Meeting.* New Orleans: The American Society of Tropical Medicine and Hygiene.

[32] Kibugu JK, Ngeranwa JJ, Makumi JN, Gathumbi JK, Kagira JM, Mwangi JN, Muchiri MW, Mdachi RE 2009 Aggravation of pathogenesis mediated by ochratoxin A in mice infected with *Trypanosoma brucei rhodesiense*. Parasitology 136, 273–281. (doi:10.1017/S0031182008005386) 19154650 10.1017/S0031182008005386

[33] Adejinmi JO, Sadiq NA, Fashanu SO, Lasisi OT, Ekundayo S 2004 Study on the blood parasite of sheep in Ibadan, Nigeria. Afr. J. Biomed. Res. 7, 42–43. (doi:10.4314/ajbr.v7i1.54066)

[34] Dubuc C, Allen WL, Maestripieri D, Higham JP 2014 Is male rhesus macaque red color ornamentation attractive to females? Behav. Ecol. Sociobiol. 68, 1215 (doi:10.1007/s00265-014-1732-9) 25246728 10.1007/s00265-014-1732-9 PMC4167843

[35] Stephen ID, Coetzee V, Law Smith MJ, Perrett DI 2009 Skin blood perfusion and oxygenation color affect perceived human health. PLoS ONE 4, e5083 (doi:10.1371/journal.pone.0005083) 19337378 10.1371/journal.pone.0005083 PMC2659803

[36] Dixson AF 1983 Observations on the evolution and behavioral significance of ‘sexual skin’ in female primates. Adv. Stud. Behav. 13, 63–106. (doi:10.1016/S0065-3454(08)60286-7)

[37] Fontaine R 1981 The uakaris, genus *Cacajao*. In *Ecology and behavior of neotropical primates* (eds AF Coimbra-Filho, RA Mittermeier), pp. 443–493. Rio de Janeiro, Brazil: Academia Brasileira de Ciencias.

[38] Trivers RL 1972 Parental investment and sexual selection, 1871-1971. In *Sexual selection and the descent of man* (ed. B Campbell), pp. 136–179. London, UK: Heinemann Educational Publishers.

